# Integrating multiple data sources to predict all-cause readmission or mortality in patients with substance misuse

**DOI:** 10.1371/journal.pdig.0001008

**Published:** 2025-09-18

**Authors:** Tim Gruenloh, Preeti Gupta, Askar Safipour Afshar, Madeline Oguss, Elizabeth Salisbury-Afshar, Marie Pisani, Ryan P. Westergaard, Michael Spigner, Megan Gussick, Matthew Churpek, Majid Afshar, Anoop Mayampurath

**Affiliations:** 1 Department of Biostatistics and Medical Informatics, School of Medicine and Public Health, University of Wisconsin-Madison, Madison, Wisconsin, United States of America; 2 Department of Medicine, School of Medicine and Public Health, University of Wisconsin-Madison, Madison, Wisconsin, United States of America; 3 Division of Pulmonary, Critical Care, Sleep and Allergy, University of Illinois Chicago, Chicago, Illinois, United States of America; 4 BerbeeWalsh Department of Emergency Medicine, School of Medicine and Public Health, University of Wisconsin-Madison, Madison, Wisconsin, United States of America; The University of Sheffield, UNITED KINGDOM OF GREAT BRITAIN AND NORTHERN IRELAND

## Abstract

Patients with substance misuse who are admitted to the hospital are at heightened risk for adverse outcomes, such as readmission and death. This study aims to develop methods to identify at-risk patients to facilitate timely interventions that can improve outcomes and optimize healthcare resources. To accomplish this, we leveraged the Substance Misuse Data Commons to predict 30-day death or readmission from hospital discharge in patients with substance misuse. We explored several machine learning algorithms and approaches to integrate information from multiple data sources, such as structured features from a patient’s electronic health record (EHR), unstructured clinical notes, socioeconomic data, and emergency medical services (EMS) data. Our gradient-boosted machine model, which combined structured EHR data, socioeconomic status, and EMS data, was the best-performing model (c-statistic 0.746 [95% CI: 0.732-0.759]), outperforming other machine learning methods and structured data source combinations. The addition of unstructured text did not improve performance, suggesting a need for further exploration of how to leverage unstructured data effectively. Feature importance plots highlighted the importance of prior hospital and EMS encounters and discharge disposition in predicting our primary outcome. In conclusion, we integrated multiple data sources that offer complementary information from data sources beyond the typically used EHRs for risk assessment in patients with substance misuse.

## Author summary

Our study leverages information from diverse data sources to develop predictive models to identify patients with substance use who are at increased risk of adverse outcomes, such as readmission and death, within 30 days of hospital discharge. We found that a gradient-boosted machine learning model that combined structured electronic health record data, socioeconomic status, and ambulance data achieved the highest performance in predicting these outcomes among patients with substance misuse. Multimodal non-deep and deep learning approaches designed to incorporate unstructured clinical notes did not enhance model performance. However, feature importance analysis revealed that prior ambulance encounters are a key predictor for our model, underlining the importance of using multiple data sources in clinical decision-making for patients with substance misuse.

## Introduction

Substance misuse, including the use of illicit substances and the use of non-illicit substances above recommended limits, is a multifaceted and complex problem that affects many individuals as well as society. Mortality from opioid misuse, non-opioid illicit use (i.e., cocaine, methamphetamine), and alcohol misuse continue to rise per year, with drug overdose-related mortality remaining at very high levels [1,2]. Substance misuse is also a leading cause of hospital readmissions [3]. Recognizing its substantially detrimental impact, hospitals deploy tertiary prevention efforts that seek to minimize morbidity and mortality associated with substance misuse [4]. For example, engaging support from peer recovery groups and addiction medicine consult services for a patient during hospital discharge has been associated with improved patient outcomes [5–9]. Because the substance misuse epidemic has continued, the Office of National Drug Control Policy (ONDCP) recently recommended broadening the focus of tertiary efforts to prevent warning signs, e.g., hospital encounters, that precede fatal events [10]. Yet, current tertiary prevention efforts are misaligned with ONDCP recommendations as they are deployed according to community-level rates of fatal events and do not consider hospitalizations that lie on the path to mortality [11]. In addition, tertiary prevention efforts are deployed based on risk assessment that is not based on relevant data. For example, a provider’s evaluation of a patient’s risk of overdose could be biased or lack critical information, such as the patient’s medical history or socioeconomic status. Therefore, risk assessment for patients with substance misuse falls short of addressing broader national policy goals by remaining focused on predicting mortality with limited data sources [12–14].

To address these gaps, we recently constructed the Substance Misuse Data Commons (SMDC), a centralized multimodal data hub hosted on a cloud-based infrastructure [15]. The SMDC integrates diverse data sources, including electronic health record (EHR) systems, emergency medical services (EMS), neighborhood-level socioeconomic status, insurance claims, and public health information, all linked securely through privacy-preserving record linkage. The SMDC allows researchers to build a comprehensive longitudinal timeline of patients with substance misuse seen at hospitals in southern Wisconsin. Therefore, the SMDC provides a unique opportunity for advancing tertiary prevention by expanding risk assessment from overdoses to future readmissions, thereby facilitating the identification of at-risk patients for interventions such as engagement with peer recovery coaches or addiction specialists, being prescribed medication for substance use disorders, or being referred to community-based treatment resources. Additionally, the SMDC allows the exploration of novel risk factors associated with outcomes among patients with substance misuse [16]. However, it remains unknown whether integrating information from SMDC sources can improve our ability to predict adverse outcomes after these patients are discharged.

The objective of this study was to develop a machine learning model capable of predicting adverse outcomes, defined as readmission or mortality, among patients with substance misuse within 30 days of hospital discharge. We hypothesize that we could improve performance in predicting post-discharge outcomes for these patients by merging EHR structured data with information from EMS data, neighborhood-level socioeconomic data, and unstructured EHR notes and reports. Bridging information from multiple data sources may lead to more accurate prioritization of patients for targeted interventions, ultimately preventing readmissions or death for patients with substance misuse.

## Results

### Study population

Out of the 28,041 encounters in the cohort, 9,362 (33%) had the primary outcome of either all-cause mortality or any hospital readmission within 30 days of discharge. Table 1 compares the clinical characteristics of encounters with and without the primary outcome. Most notably, in comparison to encounters without a primary outcome, those with the primary outcome were slightly older (median age 47 years vs. 46 years, P = 0.002), had a larger proportion of Black race (13% vs. 11%, P < 0.001), a higher proportion of leaving against medical advice (AMA) (4.6% vs. 2.3%, P < 0.001), a lower proportion of being discharged to home (73% days vs. 85% days, P < 0.001), and had at least one encounter in the prior 30 days (29% vs. 9%, P < 0.001). There were no statistically significant differences between the proportion of males (62.6% vs. 61.9%, P = 0.258) and the proportion of those admitted for an opioid-related reason (23.7% vs. 23.7%, P = 0.907) between encounters with a primary outcome and those without a primary outcome.

**Table 1:** Comparison of characteristics of patients who experience the primary outcome of 30-day rehospitalization or death after discharge against those who did not.

Variable	Patients with the primary outcome, (n = 9,362)	Patients without the primary outcome, (n = 18,679)	p-value
Age (median [IQR])	47 [34, 58]	46 [31, 59]	0.001
Male (%)	5859 (62.6)	11,559 (61.9)	0.260
Length of Stay (mean (sd))	5.16 (8.85)	4.64 (8.04)	<0.001
Unplanned Admission (%)	5,518 (58.9)	9,781 (52.4)	<0.001
Number of Encounters 30 Days Prior (%)			<0.001
0	6596 (70.5)	16906 (90.5)	<0.001
1	1375 (14.7)	1367 (7.3)	<0.001
> 1	1391 (14.9)	406 (2.2)	<0.001
Race (%)			<0.001
White	7,753 (82.8)	15,843 (84.8)	<0.001
Black	1,234 (13.2)	2,097 (11.2)	<0.001
Other	375 (4.0)	739 (4.0)	0.868
Ethnicity (%)			<0.001
Hispanic/Latino	314 (3.4)	792 (4.2)	<0.001
Not Hispanic or Latino	8,974 (95.9)	17,664 (94.6)	<0.001
Unknown	74 (0.8)	223 (1.2)	0.002
Discharge Disposition (%)			<0.001
Discharged to Facility	1,284 (13.7)	1,646 (8.8)	<0.001
Discharged to Home	6,918 (73.9)	1966 (85.5)	<0.001
Other/Unspecified	727 (7.8)	639 (3.4)	<0.001
AMA	433 (4.6)	428 (2.3)	<0.001
Comorbidities (%)			<0.001
Congestive Heart Failure	1,509 (16.1)	2,473 (13.2)	<0.001
Chronic Pulmonary Disease	4,112 (43.9)	6,735 (36.1)	<0.001
Renal Failure	1,433 (15.3)	2,237 (12.0)	<0.001
Liver Disease	4,756 (50.8)	6,074 (32.5)	<0.001
Fluid and Electrolyte Disorders	6,647 (71.0)	9,817 (52.6)	<0.001
Psychoses	1,742 (18.6)	2,079 (11.1)	<0.001
Depression	6,673 (71.3)	10,850 (58.1)	<0.001
Substance Misuse Type (%)			<0.001
Alcohol	6,084 (65.0)	11,743 (62.9)	0.001
Opioid	4,433 (23.7)	2,213 (23.6)	0.873
Cannabis	2,598 (13.9)	974 (10.4)	<0.001
Cocaine	1,046 (11.2)	1,566 (8.4)	<0.001
Hallucinogens	41 (0.4)	106 (0.6)	0.184
Psychoactive and Other	1,520 (16.2)	2,667 (14.3)	<0.001
Sedative Hypnotic	193 (2.1)	364 (1.9)	0.553
Stimulant	513 (5.5)	840 (4.5)	<0.001
Encounter Type (%)			<0.001
ED Visit or Hospital Visit	7,521 (80.3)	14,480 (77.5)	<0.001
Inpatient Hospitalization	5,354 (57.2)	11,364 (60.8)	<0.001

### Model performance

Our derivation cohort consisted of 14,082 encounters, among which 29% had the primary outcome, while our longitudinal validation cohort comprised 4,057 encounters, among which 33% had the primary outcome. Table 2 shows the validation AUCs for different combinations of our structured-only data sources and machine learning models. The XGBoost EHR model using FLWB representation of longitudinal features was the best-performing EHR-only machine learning model and was superior to the baseline model that used age and number of encounters in the prior 30 days (AUC 0.739 [95% CI 0.726-0.752] vs. 0.610 [95% CI 0.594-0.625], P < 0.001). Alternative approaches to the FLBW representations for vital signs and lab results did not improve performance (S11 Table). The inclusion of socioeconomic features (i.e., ACS, ADI, or both) did not significantly improve the performance of the EHR-only models built using the elastic net, random forest, and XGBoost models. Compared to using only EHR data, using the structured data sources led to improvements in prediction for the elastic net (AUC 0.720 [95% CI 0.707-0.734] vs. 0.710 [95% CI 0.696-0.725], P < 0.001) and XGBoost models (AUC: 0.746 [95% CI 0.732-0.759] vs. 0.739 [95% CI 0.726-0.752], P = 0.028), but not for the random forest model (AUC 0.727 [95% CI 0.713-0.740] vs. 0.724 [95% CI 0.710-0.738], P = 0.415). When using all data sources, the XGBoost model outperformed a simpler, more interpretable elastic net regression model (AUC: 0.746 [95% CI 0.732-0.759] vs. AUC 0.720 [95% CI 0.707-0.734]; P < 0.001). Expanding the data sources from EHR-only to all structured data sources demonstrated an NRI of 0.77% and 0.46% for the XGBoost model at a specificity of 80% and 70%, respectively.

**Table 2:** Model comparisons with different combinations of structured data sources. Reported values are AUC with 95% confidence intervals. The p-value compares the EHR-only XGBoost model with other XGBoost models built using combinations of other data sources.

Structured Data Source	Elastic Net AUC (95% CI)	Random Forest AUC (95% CI)	XGBoost AUC (95% CI)	p-value
Baseline	0.610 (0.594-0.625)	–	–	–
EHR	0.710 (0.696-0.725)	0.724 (0.710-0.738)	0.739 (0.726-0.752)	–
EHR + ADI	0.710 (0.696-0.724)	0.724 (0.710-0.738)	0.739 (0.725-0.752)	0.713
EHR + ACS	0.713 (0.699-0.727)	0.716 (0.702-0.730)	0.740 (0.726-0.753)	0.914
EHR + ADI + ACS	0.713 (0.699-0.727)	0.721 (0.707-0.734)	0.740 (0.726-0.753)	0.896
EHR + ADI + ACS + EMS	0.720 (0.707-0.734)	0.727 (0.713-0.740)	0.746 (0.732-0.759)	0.028

Table 3 depicts the AUCs for the multimodal models that combined structured data sources (EHR + ADI + ACS + EMS) with unstructured clinical notes and reports. None of the multimodal prediction models were superior to the single-mode XGBoost model that used structured data alone. The structured data-only XGBoost model outperformed an unstructured data-only XGBoost model developed using a BoW-based approach (AUC 0.746 [95% CI: 0.732-0.759] vs. 0.726 [95% CI: 0.713-0.740], P = 0.044). Among multimodal models, the BoW-based approach to representing unstructured text achieved the highest AUC, but the improvement in performance compared to the structured data-only XGBoost model was not statistically significant (AUC 0.754 [95% CI 0.741-0.767 vs. 0.746 [95% CI 0.732-0.759], P = 0.191). The early and joint fusion models underperformed, while the XGBoost with SapBERT-embeddings and the late-fusion deep learning model were similar in performance to the single modality structured data-only XGBoost model (see Table 3).

**Table 3:** Model comparisons for the multimodal machine learning models against each other and against the best-performing structured data-only model. The p-value compares each multimodal approach with the best-performing model on the structured data (XGBoost with EHR + ADI + ACS + EMS from Table 1).

Data and Model Type	AUC (95% CI)	P-value
Structured Data **Only XGBoost**	0.746 (0.732-0.759)	–
Structured + Unstructured Data **(BoW) XGBoost**	0.754 (0.741-0.767)	0.191
Structured + Unstructured Data **(SapBERT) XGBoost**	0.751 (0. 738-0.764)	0.300
Structured + Unstructured Data **(SapBERT) Early Fusion Deep Learning**	0.738 (0.725-0.751)	0.786
Structured + Unstructured Data **(SapBERT) Joint Fusion Deep Learning**	0.714 (0.700-0.728)	0.999
Structured + Unstructured Data **(SapBERT) Late Fusion Deep Learning**	0.749 (0.736-0.762)	0.350

AUPRC comparisons for all structured-only source combinations and for all multimodal models are shown in S12 and S13 Tables. The AUPRC comparison metrics aligned with the AUC findings, as the structured data-only XGBoost model demonstrated the best AUPRC. Comparisons of sensitivity, specificity, and positive and negative predictive values between the best-performing structured data-only XGBoost model and the baseline model are shown in S14 Table. At a fixed specificity of 70%, the structured data-only XGBoost model had a higher sensitivity (65% vs. 44%), a higher positive predictive value (55% vs. 45%), and a higher negative predictive value (78% vs. 68%) than the baseline model. Similarly, at a fixed sensitivity of 80%, the structured data-only XGBoost model had a higher specificity (64% vs. 29%), a higher positive predictive value (49% vs. 40%), and a higher negative predictive value (82% vs. 72%). S15 Table depicts the subgroup-level performance of our structured data-only XGBoost model among different substance misuse types in terms of AUC and sensitivity and specificity at the Youden’s Index. The model demonstrated better performance among patients with alcohol misuse and non-alcohol, non-opioid misuse compared to patients with opioid misuse.

**Fig 1:**
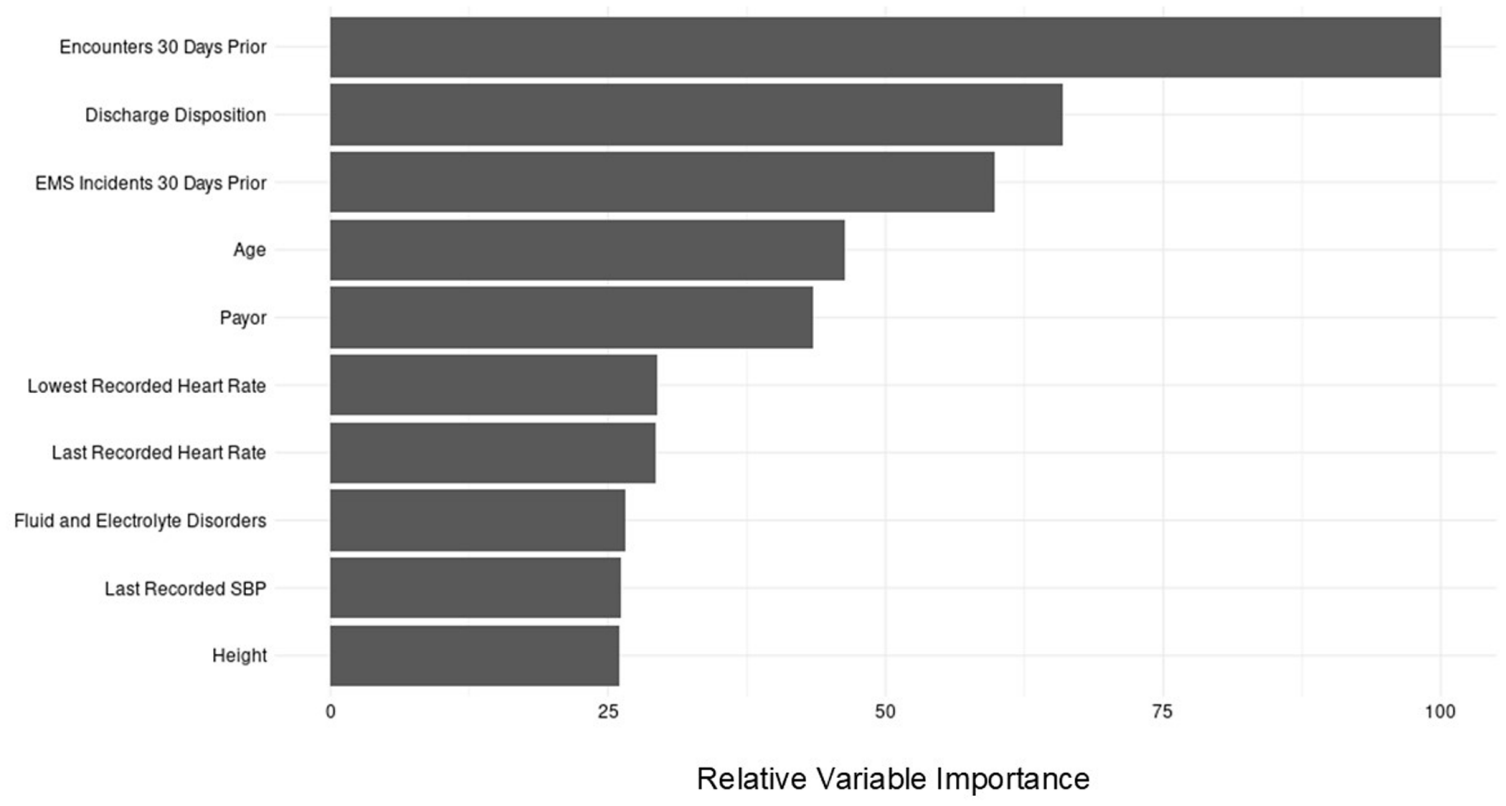
Variable importance for the structured data-only XGBoost model. EMS incidents prior to 30 days refer to EMS incidents that resulted in a patient being transported to the hospital.

Fig 1 shows the top 10 most important features for the best-performing structured-only XGBoost model incorporating EHR, ADI, ACS, and EMS data. The number of encounters in the prior 30 days emerged as the most influential predictor. Other key features included discharge disposition and EMS incidents to the hospital 30 days before the encounter. Age, insurance payor, heart rate, systolic blood pressure, and having a prior fluid and electrolyte disorder also demonstrated high importance (see Fig 1).

## Discussion

In this study, we investigated whether combining information from several data sources could improve the identification of patients with substance misuse at risk of readmission or mortality within 30 days of hospital discharge. Our gradient-boosted model, which incorporated features from the EHR, EMS, and socioeconomic structured data sources, demonstrated superior performance compared to the baseline model, models created from alternative structured data combinations, and multimodal fusion models that integrated features from structured and unstructured data sources. This predictive model can be used to enhance tertiary prevention efforts by identifying patients with substance misuse who are at risk for readmission or death, thereby facilitating timely interventions such as harm reduction services, medication for substance use disorders, linkage to community-based treatment resources, and engagement with peer recovery coaches.

National policy updates emphasize the need for hospitals and regional public health agencies to shift away from focusing solely on drug fatalities to using data from non-fatal events for designing treatment and interventions [17]. However, hospitals face significant challenges in implementing these recommendations due to the limited use and scope of current non-fatal risk assessment approaches. Most models published in literature predict deleterious events for narrow populations, such as patients of a single substance use type, and are created to operate only after an overdose event or using non-granular data. For example, Kinreich et al. developed a machine learning model that used functional brain activity, polygenic risk scores, medications, and demographic information to predict remission among patients with alcohol use disorder [14]. In another study, Morel et al. developed a model to predict 30-day readmission in patients with mental or substance use disorders but are limited to using features from insurance claims [18]. Other models have been developed to predict mortality risk for patients with substance misuse but are exclusive to survivors of opioid overdose who are Medicare beneficiaries [12,13,19]. In contrast, our model incorporates granular data sources to predict hospitalizations and deaths beyond a single healthcare system. By focusing on predicting all-cause mortality or any hospital readmission within 30 days of a hospital or ED discharge of a patient with substance misuse, our approach provides a more comprehensive risk assessment that enables current tertiary prevention efforts to better align with national policy.

Several studies have applied deep learning models to predict hospital readmission both in a general cohort [20,21] and among patients with specific conditions, such as heart disease [22] or diabetes [23]. Recent work has also explored the utility of including information from notes [24–27], with variations on text-based feature representation. For example, Loutati et al. utilized TFIDF representation [26], while Sheetrit et al. and Golmaei & Luo used representations of notes acquired from ClinicalBERT [25,28]. In contrast, Tang et al. used ontology-mapped codes from discharge notes in a spatio-temporal graph neural network [27]. Our study contributes to this body of research by exploring CUI-based methods for integrating multisource structured and unstructured data into predicting outcomes among patients with substance misuse.

Our study demonstrates the utility of using an integrated resource for outcomes prediction among patients with substance misuse. Using privacy-preserving linkage, we were able to link state-level public health data with longitudinal EHR and national mortality datasets, providing an opportunity to holistically study outcomes and conditions related to substance misuse. The future expansion of the SMDC to include patients from other hospitals is likely to further enhance model performance, as it will utilize additional information (e.g., details from prior hospitalizations at other centers) that is critical to predicting outcomes. Recent studies have highlighted limitations in using federated learning approaches for model development [29]. Our study suggests that pooling information across sources could lead to improved risk stratification in this study population.

Our study indicates that the best performance for identifying patients with substance misuse at risk of readmission or death within 30 days of hospital discharge is achieved by combining information from structured EHR features (physiological observations, comorbidities, and admission-level details) with structured EMS data. Among the machine learning models tested, the gradient-boosted machine performed the best, highlighting the model’s ability to use non-linearity and interactions among the features to improve performance.

The model’s output can be converted into a risk-based assessment of the likelihood of being readmitted or dying within 30 days of hospital discharge. This score can be the foundation of a clinical decision support tool to identify and treat patients who could benefit from targeted interventions designed to reduce subsequent harm from substance misuse. For example, based on this score crossing an operational threshold, clinicians could prescribe naloxone or initiate a consult with an addiction medicine specialist. Additionally, the patient could be referred to harm reduction or enhanced case management services, which could lead to a lower risk of overdose [30]. In particular, engagement with peer recovery groups may also improve outcomes [5]. Hospitals could further customize the choice of an operational threshold by balancing model sensitivity and specificity with the patient population and resource capacities. However, we note that our model is an example of a static model where the risk of readmission is predicted at the time of hospital discharge. Adapting to a dynamic prediction workflow that enables continuous readmission risk prediction throughout a patient’s hospital stay, similar to a recent study by Jiang et al. [31], could facilitate early intervention, particularly during the hospitalization itself.

By incorporating a broader range of structured data sources that may contain unique risk factors for our primary outcome, our model captures a more comprehensive perspective on the multifaceted complexities of substance misuse. Our variable importance plot underscores the significance of encounters before admission for predicting 30-day readmission, a finding supported by prior studies [18,32–35]. However, our model outperformed the baseline model that contained age and number of prior encounters, indicating that it uses information within other data sources to improve prediction performance. For instance, EMS information, mainly the number of EMS incidents, was a significant predictor of our outcome by being among the top features in the feature importance plot. This observation aligns with prior studies regarding the importance of using EMS data for risk assessment in this population [16]. Similarly, discharge disposition, particularly when a patient is discharged against medical advice, was noted to be an important predictor of our primary outcome, corroborating previous findings [36–38]. Additionally, insurance coverage was observed to be an important feature, supporting results from published studies that link insurance status to treatment access for patients with substance misuse [39–42]. Vital signs and comorbidities were also determined to be important to predicting our primary outcome.

Recent studies have highlighted the value of patient notes in the identification of patients with substance misuse [43]. Our study indicates that patient notes have limited utility in predicting 30-day readmission or mortality for these patients, as our multimodal prediction models could not outperform the structured data-only XGBoost model. In the general patient population, prior studies have primarily used structured data elements to predict the risk of readmission [34]. While multimodal approaches have been recently proposed to predict readmission risk in other cohorts [26], our study is the first to explore advanced multimodal architectures for predicting 30-day outcomes among patients with substance misuse. Our study is notable for extensively testing various forms of longitudinal data representations, text representations, and multimodal fusion algorithms for improving model performance.

This study has several limitations. First, it is a retrospective study conducted at a single academic hospital system, which may limit the generalizability of our findings. Our model may not perform well in patients with substance misuse from regions that differ in demographics and outcomes from our cohort. However, efforts in tertiary prevention improvements must vary by local population and health systems, and the methods in this study can be translated to other regions and states where the substance misuse epidemic also continues unabated. Studies focusing on implementation followed by prospective evaluation and validation of our model are also crucial to ensure broad clinical applicability. Second, using CUIs instead of full patient notes due to privacy limitations in sharing a model trained on notes may have resulted in a loss of contextual information captured in text narratives. Using CUIs also limited our ability to test different representative deep learning models for text-based representations of unstructured data. However, our approach overcomes variations in documentation practice between providers by mapping free-text phrases to curated medical concepts and may be more generalizable across hospitals. Additionally, recent advances in CUI representations could offer performance gains instead of our choice of SapBERT as a CUI-embedding representation [44], but this remains an area of future work. Finally, our data is limited to 2016–2021 and does not incorporate more recent trends in substance misuse rates.

In conclusion, we integrated information from multiple data sources using machine learning models to predict 30-day all-cause mortality or readmissions among patients with substance misuse. Including EMS data improved model performance compared to models that did not include this data source. The best-performing model could be incorporated as a clinical decision support tool to improve tertiary prevention efforts and reduce the harmful impact of substance misuse by enabling them to be better aligned with national policy recommendations.

## Materials and methods

### Study population

As described in our previous study, the SMDC comprises 32,522 patients with 64,171 encounters (defined as emergency department (ED) visits or inpatient hospitalizations) across two University of Wisconsin (UW) academic hospitals [15]. Based on the completeness of EMS data, patients in the SMDC who had at least one hospital encounter between January 1, 2017, and December 31, 2021, were included in the study cohort, resulting in 28,762 encounters. The main exclusion criteria were hospital encounters with in-hospital deaths, resulting in 28,041 available encounters for predicting the primary outcome. Only those encounters with available structured and unstructured EHR data were considered, which resulted in a final cohort of 28,039 encounters. The UW-Madison Health Sciences Internal Review Board (IRB) approved using the SMDC data as a Health Insurance Portability and Accountability Act (HIPAA)-limited (i.e., with patient identifiers removed but with dates, timestamps, and census block group retained) dataset for this study with a waiver of informed consent. (IRB# 2021–0553)

### Outcome

The primary outcome of interest in this study was a composite event of all-cause mortality or unplanned readmission within 30 days after the discharge date of an encounter. All-cause mortality was extracted from three sources within the SMDC: (1) future EHR encounters with in-hospital death or recorded known out-of-hospital death; (2) state Vital Statistics, which is a collection of death certificates maintained by the state’s Department of Health Services; and (3) a commercial national data source provided by Datavant Solution that included deaths from the Social Security Administration’s Death Master File augmented with deaths sourced from funeral homes and newspaper obituary reports. Readmissions were identified using data within our EHR system (i.e., readmissions to our hospital) as well as statewide insurance claims (i.e., readmissions outside of our hospital) that were obtained from the Wisconsin Health Information Organization (WHIO), a claims organization with 75% catchment of insurance claims, including Medicaid and Medicare claims, from 4.5M insured persons residing in Wisconsin. We utilized the Center for Medicare & Medicaid Services definition of unplanned hospital readmissions, which excludes expected readmissions to the same hospital or another applicable acute care hospital within 30 days of discharge from the original admission [45].

### Model features

Features were constructed from the EHR, neighborhood-level socioeconomic data sources, and EMS datasets within the SMDC. Below are details of various feature engineering approaches. The complete list of features can be found in S1–S8 Tables.

EHR-based Features: Data related to the patient’s encounter, including vital signs and laboratory measurements (S1 and S2 Tables), demographics and substance-related data (S3 Table), encounter-related data (e.g., intensive care unit admission, means of arrival, number of encounters in prior 30 days, see S4 Table), and prior comorbidities (S5 Table) were extracted from the SMDC. Longitudinal EHR data observations of vital signs and laboratory results were represented using the first, last, worst, and best (FLWB) values, where the worst and best notations for each feature were based on expert review (MA). Two alternative approaches to the data representation were evaluated: (1) by extracting summary time series features (e.g., slope, autocorrelation) using the tsfresh Python package [46], and (2) by encoding values using a decision-tree-based piecewise linear encoder [47].

Socioeconomic Features: The American Community Survey (ACS, S6 Table) census bureau data was included to account for neighborhood-level socioeconomic factors within each patient’s census block. The Area Deprivation Index (ADI, S7 Table) national and state ranking of disadvantage was also included as an additional measure of a patient’s socioeconomic status, which has been associated with hospital readmission in other studies [48–50].

EMS Features: Features related to a patient’s ambulance visits record to any hospital (i.e., not limited to UW hospitals) from the EMS data source within the SMDC (see S8 Table). The number of EMS incidents within the prior 30 days of the encounter was used as an EMS-related feature. We included EMS incidents that transported the patient to the hospital and those that did not transport the patient to the hospital as separate features. We excluded EMS incidents within 24 hours before an encounter as these are most likely associated with that encounter. Additionally, EMS data associated with an encounter was concatenated to the EHR data as observations (e.g., the blood pressure obtained by EMS was the first observation in a contiguous series of prehospital and hospital blood pressure measurements).

Text-based Features from Patient Notes and Reports: The full corpus of documents from the EHR was included. All notes collected during the patient’s hospital stay were preprocessed using an open-source natural language processing engine, called the clinical Text And Knowledge Extraction System (cTAKES) [51], to convert raw text into coded medical concepts, called Concept Unique Identifiers (CUIs), which are sourced from the National Library of Medicine Metathesaurus. CUIs that occurred 20 or fewer times in all encounters were removed. The final vocabulary size was 18,332 CUIs, which served as input features for the prediction model.

We evaluated two representations of CUIs for our prediction models. First, Term Frequency-Inverse Document Frequency (TF-IDF) metrics for CUIs were used to create a bag of words (BoW)-based representation of features for input to the model. Second, a high-dimensional representation embedding of CUIs using SapBERT was created [52]. Briefly, the preferred text for all CUIs for an index hospitalization was combined into a single document and given as input to SapBERT. This results in a representation such that CUIs with similar meanings will be close together in the SapBERT embedded space, allowing models to be aware of the semantics between two CUIs. To obtain a representation that traditional machine learning models can use, we took the max value of each dimension, i.e., max-pooling, of the embedding over all CUIs in the encounter. Other text-based embedding representation methods could not be tested since our unstructured data consisted of CUIs.

### Model development

**Fig 2:**
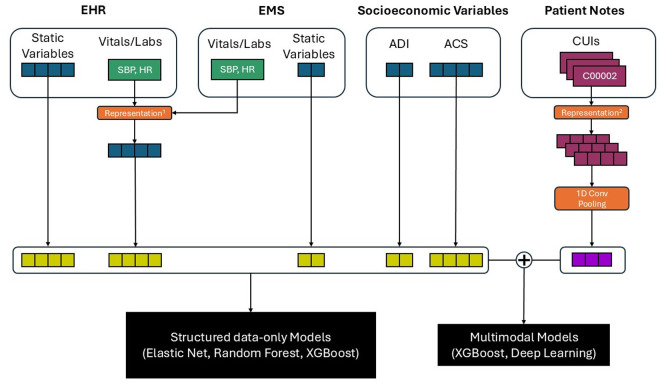
Flowchart of study design depicting processing of heterogenous data and machine learning models. Representation^1^ method indicates selecting the first, last, worst, and best values from the time series EHR data. Representation^2^ methods include CUI-based representations for clinical text.

Fig 2 illustrates the study workflow. Data were split longitudinally into model derivation (encounters admitted between 2017 and 2020) and model validation (encounters in 2021) cohorts. Using the structured data sources, elastic net, random forest, and gradient-boosted machine (XGBoost) algorithms were applied to develop 15 machine learning models based on the following combinations: (1) EHR only, (2) EHR and ADI, (3) EHR and ACS, (4) EHR, ADI, and ACS, and (5) EHR, ADI, ACS, and EMS. The initial goal was to identify the best-performing and most parsimonious model from the structured data.

In addition, two multimodal XGBoost machine learning models were developed, integrating structured data features with text-based features (either BoW- or SapBERT embedding-based representations) extracted from unstructured clinical notes and reports. Three deep-learning multimodal architectures that combined structured and unstructured data using early, joint, and late fusion strategies [53,54], were also created (see S1 Fig for workflow). The early fusion approach passes the SapBERT-based embeddings through a single convolution layer, after which the structured features are concatenated and fed into a dense neural network layer for prediction. For the joint fusion approach, a structured data representation was first created using a dense layer and then fused with a convolutional neural network (CNN)-based embedding representation of the clinical text. CNN-based embeddings were obtained using a 1-dimensional convolution over all SapBERT-embeddings within the index encounter. This approach allowed us to use all CUI information instead of the max-pooled approach. The late fusion model used a combination of XGBoost and a CNN to make predictions using structured and clinical text, respectively. These two predictions were fed into a logistic regression to make the final prediction of our primary outcome.

Finally, since prior admissions strongly predict readmissions [18,32–35], a baseline elastic net model was developed using only patient age and the number of previous encounters within 30 days. Hyperparameter optimization was performed using a grid search for all our machine learning models. The full list of hyperparameters optimized for each model is shown in S9 and S10 Tables.

### Model performance

The primary metric for assessing model performance was the Area Under the Receiver Operating Characteristic Curve (AUC). AUC comparisons between models were performed using Delong’s method [55]. The secondary metric was the Area Under the Precision-Recall Curve (AUPRC) for all models. The Absolute Net Reclassification Index (NRI) was used to measure the additive benefit from adding non-EHR structured data sources compared to the EHR-only model [56]. Sensitivity, specificity, and positive and negative predictive values were compared between the baseline and best-performing model. Additionally, subgroup analyses of model performance by substance type were further examined (alcohol vs. opioid vs. other) by comparing AUC and sensitivity/specificity at the Youden’s Index. Finally, we analyzed the importance of features, calculated using information gain, of the best-performing model.

## Supporting information

S1 Table A list of features – Vitals. (S1_Table.DOCX)

S2 Table A list of features – Labs. (S2_Table.DOCX)

S3 Table A list of features – Demographic and Substance Misuse Related Information. (S3_Table.DOCX)

S4 Table A list of features – Encounter-specific information. (S4_Table.DOCX)

S5 Table A list of features – Prior Comorbidities. ICD-based prior comorbidities.
(S5_Table.DOCX)

S6 Table A list of features – ACS. The American Community Survey (ACS) collects data on what social, economic, housing, and demographic changes are taking place in their communities.
(S6_Table.DOCX)

S7 Table A list of features – ADI. The Area Deprivation Index, also known as ADI, is a neighborhood socioeconomic disadvantage index created by the Health Resources and Services Administration and maintained by the Neighborhood Atlas at the University of Wisconsin-Madison. ADI measures the level of socioeconomic disadvantage in neighborhoods. ADI was created by a factor analysis of the ACS variables. The ADI State variable allows the comparison of neighborhoods at the state level. The ADI National variable allows the comparison of neighborhoods at the national level.
(S7_Table.DOCX)

S8 Table A list of features – EMS. (S8_Table.DOCX)

S9 Table Hyperparameters for Non-Deep Learning Methods. (S9_Table.DOCX)

S10 Table Hyper-parameters for Deep Learning Fusion Models. (S10_Table.DOCX)

S11 Table Comparison of longitudinal data representations. Comparing three ways of representing time series EHR vital signs and laboratory values (first, last, worst, and best value, extracting time series features using the tsfresh Python package, and using a piecewise linear encoder) by evaluating performance on an elastic net model.
(S11_Table.DOCX)

S12 Table AUPRC for models using structured data only. AUPRC 95% confidence intervals are calculated by bootstrapping.
(S12_Table.DOCX)

S13 Table AUPRC for multimodal prediction models compared to models that used structured data only. AUPRC 95% confidence intervals are calculated by bootstrapping.
(S13_Table.DOCX)

S14 Table Sensitivity and specificity analysis. (S14_Table.DOCX)

S15 Table Structured data-only XGBoost Model performance by substance type. *Other refers to not alcohol and not opiate.
(S15_Table.DOCX)

S1 Fig Illustration of our deep learning multimodal architectures. (a) The early-fusion approach concatenated variables from the structured-only data sources with the convolutional layer output to which the SapBERT-based embeddings are fed. (b) The joint-fusion approach combined a dense layer representation of structured features with CNN-based embedding of clinical text. (c) The late fusion approach combined predictions from an individually trained XGB model (created from structured data) and a CNN model (created from text-based features) and fed them into a logistic regression (LR) model to make the final prediction.
(S1_Fig.DOCX)
