# Supplementary material for: Integrating multiple data sources to predict all-cause readmission or mortality in patients with substance misuse
Source: PLOS Digit Health. 2025 Sep 18;4(9):e0001008. doi: 10.1371/journal.pdig.0001008 (PMC12445462; doi:10.1371/journal.pdig.0001008)
Supplement: S1 Table — (S1_Table.DOCX) [file pdig.0001008.s001.docx]

**S1 Table: A list of features – Vitals.**

| Vitals Taken |
| --- |
| AVPU |
| Diastolic blood pressure |
| Heart rate |
| Oxygen flow |
| Oxygen saturation (SpO2) |
| Respiratory rate |
| Systolic blood pressure |
| Temperature |
| Height |
| Weight |
| BMI |
