# Supplementary material for: Integrating multiple data sources to predict all-cause readmission or mortality in patients with substance misuse
Source: PLOS Digit Health. 2025 Sep 18;4(9):e0001008. doi: 10.1371/journal.pdig.0001008 (PMC12445462; doi:10.1371/journal.pdig.0001008)
Supplement: S2 Table — (S2_Table.DOCX) [file pdig.0001008.s002.docx]

**S2 Table: A list of features – Labs.**

| Lab Measurements |
| --- |
| Albumin level |
| Alcohol level |
| Alcohol (binary) |
| Alkaline phosphatase level |
| Ammonia level |
| Urine or serum drug test was positive for amphetamines |
| Immature neutrophils level |
| Absolute basophil count levels |
| Percentage of basophils |
| Urine or serum drug test was positive for benzodiazepines |
| Total bilirubin level |
| Absolute blast cell level |
| Percentage of blast cells |
| Blood urea nitrogen level |
| Calcium level |
| Chloride level |
| Carbon Dioxide level |
| Urine or serum drug test was positive for cocaine |
| Creatinine level |
| C reactive protein level |
| D-Dimer level |
| If a urine or serum drug test was done |
| Absolute eosinophils level |
| Percentage of eosinophils |
| Erythrocyte sedimentation rate |
| Ferritin level |
| Fibrinogen level |
| Glucose level |
| Hemoglobin level |
| INR (international normalized ratio) level |
| Lactate level |
| Lactate dehydrogenase level |
| Lipase level |
| Absolute Lymphocytes level |
| Lymphocyte percentage |
| Magnesium level |
| Mean Corpuscular Volume |
| Absolute Monocytes level |
| Monocyte percentage |
| Absolute Neutrophils level |
| Neutrophil percentage |
| Urine or serum drug test was positive for opiates |
| Partial pressure of carbon dioxide in arterial blood |
| Partial pressure of carbon dioxide in venous blood |
| Arterial pH |
| Venous pH |
| Phosphate level |
| Platelet count |
| Partial pressure of oxygen in arterial blood |
| Partial pressure of oxygen in venous blood |
| Potassium level |
| Procalcitonin level |
| Partial Thromboplastin Time test level |
| Red Cell Distribution Width test level |
| Aspartate minotransferase (AST) level |
| Alanine Aminotransferase (ALT) level |
| Sodium level |
| Total protein level |
| Troponin level |
| White blood cell count |
