# Supplementary material for: Integrating multiple data sources to predict all-cause readmission or mortality in patients with substance misuse
Source: PLOS Digit Health. 2025 Sep 18;4(9):e0001008. doi: 10.1371/journal.pdig.0001008 (PMC12445462; doi:10.1371/journal.pdig.0001008)
Supplement: S3 Table — (S3_Table.DOCX) [file pdig.0001008.s003.docx]

**S3 Table: A list of features – Demographic and Substance Misuse Related Information.**

| Encounter Information |
| --- |
| Alcohol use based on ICD code |
| Cannabis used based on ICD code |
| Cocaine use based on ICD code |
| Hallucinogen use based on ICD code |
| Opioid use based on ICD code |
| Psychoactive and other substance use based on ICD code |
| Sedative/hypnotic substance use based on ICD code |
| Stimulant use based on ICD code |
| No substance use ICD code |
| Patient was treated in the ED |
| Patient had an inpatient hospital stay |
| Patient was in prison during the time of the encounter |
| Age in years |
| Self-report sex at birth by patient |
