# Supplementary material for: Integrating multiple data sources to predict all-cause readmission or mortality in patients with substance misuse
Source: PLOS Digit Health. 2025 Sep 18;4(9):e0001008. doi: 10.1371/journal.pdig.0001008 (PMC12445462; doi:10.1371/journal.pdig.0001008)
Supplement: S4 Table — (S4_Table.DOCX) [file pdig.0001008.s004.docx]

**S4 Table: A list of features – Encounter-specific information.**

| Hospital Features |
| --- |
| The number of encounters 30 days prior to the current encounter |
| Length of Stay of Encounter in Days |
| Discharge Disposition |
| First service after Emergency Medicine, if patient had more than one service during encounter |
| Last service of encounter |
| Patient visited ED during encounter |
| Patient visited OR during encounter |
| The patient was in a procedure or intervention during encounter |
| Patient was in a general ward during encounter |
| Patient was in the ICU during encounter |
| Patient was in an IMC during encounter |
| Hospital site |
| Means of arrival to the hospital |
| Payor class |
