# Supplementary material for: Integrating multiple data sources to predict all-cause readmission or mortality in patients with substance misuse
Source: PLOS Digit Health. 2025 Sep 18;4(9):e0001008. doi: 10.1371/journal.pdig.0001008 (PMC12445462; doi:10.1371/journal.pdig.0001008)
Supplement: S5 Table — ICD-based prior comorbidities. (S5_Table.DOCX) [file pdig.0001008.s005.docx]

**S5 Table: A list of features – Prior Comorbidities.** ICD-based prior comorbidities.

| Comorbidities |
| --- |
| The patient had a diagnosis of congestive heart failure |
| The patient had a diagnosis of a cardiac arrhythmia |
| The patient had a diagnosis of a valvular disease |
| The patient had a diagnosis of a pulmonary circulation disorder |
| The patient had a diagnosis of a peripheral vascular disorder |
| The patient had a diagnosis of hypertension, uncomplicated |
| The patient had a diagnosis of hypertension, complicated |
| The patient had a diagnosis of paralysis |
| The patient had a diagnosis of other neurological disorder |
| The patient had a diagnosis of chronic pulmonary disease |
| The patient had a diagnosis of diabetes, uncomplicated |
| The patient had a diagnosis of diabetes, complicated |
| The patient had a diagnosis of hypothyroidism |
| The patient had a diagnosis of renal failure |
| The patient had a diagnosis of liver disease |
| The patient had a diagnosis of peptic ulcer disease, excluding bleeding |
| The patient had a diagnosis of AIDS/HIV |
| The patient had a diagnosis of lymphoma |
| The patient had a diagnosis of metastatic cancer |
| The patient had a diagnosis of solid tumor without metastasis |
| The patient had a diagnosis of rheumatoid arthritis or collagen vascular disease |
| The patient had a diagnosis of coagulopathy |
| The patient had a diagnosis of obesity |
| The patient had a diagnosis of weight loss |
| The patient had a diagnosis of fluid and electrolyte disorders |
| The patient had a diagnosis of blood loss anemia |
| The patient had a diagnosis of deficiency anemia |
| The patient had a diagnosis of alcohol misuse |
| The patient had a diagnosis of drug misuse |
| The patient had a diagnosis of psychosis |
| The patient had a diagnosis of depression |
