# Supplementary material for: Integrating multiple data sources to predict all-cause readmission or mortality in patients with substance misuse
Source: PLOS Digit Health. 2025 Sep 18;4(9):e0001008. doi: 10.1371/journal.pdig.0001008 (PMC12445462; doi:10.1371/journal.pdig.0001008)
Supplement: S6 Table — The American Community Survey (ACS) collects data on what social, economic, housing, and demographic changes are taking place in their communities. (S6_Table.DOCX) [file pdig.0001008.s006.docx]

**S6 Table: A list of features – ACS.** The American Community Survey (ACS) collects data on what social, economic, housing, and demographic changes are taking place in their communities.

| American Community Survey |
| --- |
| Proportion of people in the patient’s neighborhood with no health insurance |
| Proportion of people in the patient’s neighborhood born in the United States |
| Proportion of people in the patient’s neighborhood who are born in a foreign country |
| Proportion of non-United States citizens in the patient’s neighborhood |
| Proportion of people in the patient’s neighborhood who are married |
| Proportion of people in the patient’s neighborhood who have never married |
| Proportion of people in the patient’s neighborhood who are divorced |
| Proportion of people in the patient’s neighborhood who are widowed |
| Proportion of people in the patient’s neighborhood under 5 years old |
| Proportion of people in the patient’s neighborhood ages 5-19 |
| Proportion of people in the patient’s neighborhood ages 20-24 |
| Proportion of people in the patient’s neighborhood ages 25-34 |
| Proportion of people in the patient’s neighborhood ages 35-44 |
| Proportion of people in the patient’s neighborhood ages 45-54 |
| Proportion of people in the patient’s neighborhood ages 55-64 |
| Proportion of people in the patient’s neighborhood ages 65-74 |
| Proportion of people in the patient’s neighborhood ages 75-84 |
| Proportion of people in the patient’s neighborhood ages 85+ |
| Proportion of people in the patient’s neighborhood who are Black |
| Proportion of people in the patient’s neighborhood who are White |
| Proportion of people in the patient’s neighborhood who are Asian |
| Proportion of people in the patient’s neighborhood who are Hispanic |
| Proportion of residents in the area who are non-Hispanic white ethnicity |
| Proportion of residents in the area who are non-Hispanic black ethnicity |
| Proportion of residents in the area who are non-Hispanic white ethnicity |
| Proportion of residents in the area who are Hispanic Census black ethnicity |
| Per Capital Income in the patient’s neighborhood |
| Proportion of fluent English speakers in the patient’s neighborhood |
| Proportion of fluent Spanish speakers in the patient’s neighborhood |
| Proportion of poor English speakers in the patient’s neighborhood |
| Proportion of people who speak another language in the patient’s neighborhood |
| Proportion of people who are employed in the patient’s neighborhood |
| Proportion of people who are unemployed in the patient’s neighborhood |
| Proportion of people who are not in the labor force in the patient’s neighborhood |
| Proportion of people who are Veterans in the patient’s neighborhood |
| Proportion of people who have not completed High School in the patient’s neighborhood |
| Proportion of people who are High School graduates in the patient’s neighborhood |
| Proportion of people who attended College but did not graduate in the patient’s neighborhood |
| Proportion of people who are College graduates in the patient’s neighborhood |
| Proportion of people who are in poverty in the patient’s neighborhood |
| Proportion of people who use Food Stamps in the patient’s neighborhood |
| Proportion of people who are Part-Time employees in the patient’s neighborhood |
| Proportion of people who are Full-Time employees in the patient’s neighborhood |
| Median Gross Rent in the patient’s neighborhood |
| Proportion of people who have a household size of 1 in the patient’s neighborhood |
| Proportion of people who have a household size of 2 in the patient’s neighborhood |
| Proportion of people who have a household size of 3 in the patient’s neighborhood |
| Proportion of people who have a household size of 4+ in the patient’s neighborhood |
| Total population in the patient’s neighborhood |
| Proportion of people who are female in the patient’s neighborhood |
| Proportion of people who are disabled in the patient’s neighborhood |
| Median Earnings in the patient’s neighborhood |
| Proportion of people who are homeowners in the patient’s neighborhood |
| Proportion of people who are on Medicare or Medicaid in the patient’s neighborhood |
| Proportion of people who are employers in the patient’s neighborhood |
| Direct Population randomization observation process (PROP) assessment method in the patient’s neighborhood |
| Proportion of people who are on Medicare in the patient’s neighborhood |
| Proportion of people who are on Tricare (VA) in the patient’s neighborhood |
| Median Household income in the patient’s neighborhood |
