# Supplementary material for: Integrating multiple data sources to predict all-cause readmission or mortality in patients with substance misuse
Source: PLOS Digit Health. 2025 Sep 18;4(9):e0001008. doi: 10.1371/journal.pdig.0001008 (PMC12445462; doi:10.1371/journal.pdig.0001008)
Supplement: S7 Table — The Area Deprivation Index, also known as ADI, is a neighborhood socioeconomic disadvantage index created by the Health Resources and Services Administration and maintained by the Neighborhood Atlas at the University of Wisconsin-Madison. ADI measures the level of socioeconomic disadvantage in neighborhoods. ADI was created by a factor analysis of the ACS variables. The ADI State variable allows the comparison of neighborhoods at the state level. The ADI National variable allows the comparison of neighborhoods at the national level. (S7_Table.DOCX) [file pdig.0001008.s007.docx]

**S7 Table: A list of features – ADI.** The Area Deprivation Index, also known as ADI, is a neighborhood socioeconomic disadvantage index created by the Health Resources and Services Administration and maintained by the Neighborhood Atlas at the University of Wisconsin-Madison. ADI measures the level of socioeconomic disadvantage in neighborhoods. ADI was created by a factor analysis of the ACS variables. The ADI State variable allows the comparison of neighborhoods at the state level.  The ADI National variable allows the comparison of neighborhoods at the national level.

| American Deprivation Index |
| --- |
| ADI national variable |
| ADI state variable |
