# Supplementary material for: Integrating multiple data sources to predict all-cause readmission or mortality in patients with substance misuse
Source: PLOS Digit Health. 2025 Sep 18;4(9):e0001008. doi: 10.1371/journal.pdig.0001008 (PMC12445462; doi:10.1371/journal.pdig.0001008)
Supplement: S8 Table — (S8_Table.DOCX) [file pdig.0001008.s008.docx]

**S8 Table: A list of features – EMS.**

| Emergency Medical Services |
| --- |
| Did the patient arrive to the current encounter by EMS |
| The number of EMS incidents 1-30 days prior to the encounter that went to a hospital |
| The number of EMS incidents 1-30 days prior to the encounter that did not travel to a hospital |
| Patient’s body temperature was taken |
| The patient's systolic blood pressure |
| The patient's diastolic blood pressure |
| The patient's heart rate expressed as a number per minute |
| The patient's oxygen saturation |
| The patient's respiratory rate expressed as a number per minute |
| AVPU |
