# Supplementary material for: Integrating multiple data sources to predict all-cause readmission or mortality in patients with substance misuse
Source: PLOS Digit Health. 2025 Sep 18;4(9):e0001008. doi: 10.1371/journal.pdig.0001008 (PMC12445462; doi:10.1371/journal.pdig.0001008)
Supplement: S9 Table — (S9_Table.DOCX) [file pdig.0001008.s009.docx]

**S9 Table: Hyperparameters for Non-Deep Learning Methods.**

| **Model** | **Hyper-parameters** | **Search Values** |
| --- | --- | --- |
| Elastic Net | Regularization Hyper-parameter | 0, 0.0001, 0.0003, 0.001, 0.005, 0.01, 0.1, 0.5, 1 |
|  | Mixing Hyper-parameter | 0, 0.01, 0.02, 0.5, 0.7, 1 |
| Random Forest | # of Variables Selected for Tree Growth | 100, 500, 1000 |
|  | Number of Trees | All integers in {Sqrt(# features) +/- 3} |
| XGBoost | Number of Trees | 100, 500, 1000 |
|  | Tree Depth | 2, 5, 10 |
|  | Learning Rate | 0.01, 0.1, 0.3, 0.5 |
|  | Fraction of Variables Selected for Tree Growth | 0.5, 0.7 |
|  | Fraction of Training Data Selected for Tree Growth | 0.7, 1 |
