# Supplementary material for: Integrating multiple data sources to predict all-cause readmission or mortality in patients with substance misuse
Source: PLOS Digit Health. 2025 Sep 18;4(9):e0001008. doi: 10.1371/journal.pdig.0001008 (PMC12445462; doi:10.1371/journal.pdig.0001008)
Supplement: S10 Table — (S10_Table.DOCX) [file pdig.0001008.s010.docx]

**S10 Table: Hyper-parameters for Deep Learning Fusion Models.**

| **Model** | **Hyper-parameters** | **Search Values** | **Best** |
| --- | --- | --- | --- |
| XGBoost– BoW/SapBERT | Number of Trees | 100, 500, 1000 | 1000 |
|  | Tree Depth | 2, 5, 10 | 5 |
|  | Learning Rate | 0.01, 0.1, 0.3, 0.5 | 0.01 |
|  | Fraction of Variables Selected for Tree Growth | 0.5, 0.7 | 0.5 |
|  | Fraction of Training Data Selected for Tree Growth | 0.7, 1 | 0.7 |
| Early Fusion - CNN | Learning Rate | 0.001, 0.0001, 0.00001 | 0.0001 |
|  | Batch Size | 16, 32, 64 | 64 |
|  | Kernel Size | 1, 3, 5 | 3 |
|  | Number of Kernels | 32, 64 | 64 |
|  | Dropout | 0.2, 0.3, 0.5, 0.7 | 0.5 |
|  | Number of Layers (MLP) | 2, 3, 4 | 4 |
|  | Use Learning Rate Decay | [True, False] | False |
|  | Learning Rate Decay | 0.01, 0.1, 0.5 | - |
|  | Learning Rate Step Size | 5, 10, 20 | - |
| Intermediate Fusion – CNN, MLP | Learning Rate | 0.0001 | 0.0001 |
|  | Batch Size | 32, 64 | 64 |
|  | Kernel Size | 3, 5 | 3 |
|  | Number of Kernels | 32, 64 | 64 |
|  | Dropout (Conv) | 0.2, 0.3, 0.5 | 0.3 |
|  | Dropout (EHR MLP) | 0.5, 0.7 | 0.7 |
|  | Number of Layers (MLP) | 2, 3, 4 | 3 |
|  | Use Learning Rate Decay | [True, False] | True |
|  | Learning Rate Decay | 0.01, 0.1, 0.5 | 0.5 |
|  | Learning Rate Step Size | 5, 10 | 5 |
|  | EHR MLP Layers | 1 | 1 |
|  | EHR MLP Number of Nodes | 64, 100, 1000 | 64 |
| Late Fusion – XGBoost – CNN | Number of Trees | 100, 500, 1000 | 500 |
|  | Tree Depth | 2, 5, 10 | 10 |
|  | Learning Rate | 0.01, 0.1, 0.3, 0.5 | 0.01 |
|  | Fraction of Variables Selected for Tree Growth | 0.5, 0.7 | 0.5 |
|  | Fraction of Training Data Selected for Tree Growth | 0.7, 1 | 0.7 |
|  | Learning Rate | 0.0001 | 0.0001 |
|  | Batch Size | 64 | 64 |
|  | Kernel Size | 3, 5 | 5 |
|  | Number of Kernels | 32, 64 | 64 |
|  | Dropout | 0.2, 0.3, 0.5, 0.7 | 0.3 |
|  | Number of Layers (MLP) | 2, 3, 4 | 3 |
|  | Use Learning Rate Decay | [True, False] | True |
|  | Learning Rate Decay | 0.5 | 0.5 |
|  | Learning Rate Step Size | 5 | 5 |
