# Supplementary material for: Integrating multiple data sources to predict all-cause readmission or mortality in patients with substance misuse
Source: PLOS Digit Health. 2025 Sep 18;4(9):e0001008. doi: 10.1371/journal.pdig.0001008 (PMC12445462; doi:10.1371/journal.pdig.0001008)
Supplement: S11 Table — Comparing three ways of representing time series EHR vital signs and laboratory values (first, last, worst, and best value, extracting time series features using the tsfresh Python package, and using a piecewise linear encoder) by evaluating performance on an elastic net model. (S11_Table.DOCX) [file pdig.0001008.s011.docx]

**S11 Table:** **Comparison of longitudinal data representations.** Comparing three ways of representing time series EHR vital signs and laboratory values (first, last, worst, and best value, extracting time series features using the tsfresh Python package, and using a piecewise linear encoder) by evaluating performance on an elastic net model.

| **Embedding Method** | **AUCs (95% CI)** |
| --- | --- |
| First, Last, Worst, Best | 0.710 (0.696-0.725) |
| Longitudinal summary features calculated using tsfresh Python package | 0.707 (0.693-0.722) |
| Encoded representations using the piecewise linear encoder | 0.714 (0.700-0.728) |
