# Supplementary material for: Integrating multiple data sources to predict all-cause readmission or mortality in patients with substance misuse
Source: PLOS Digit Health. 2025 Sep 18;4(9):e0001008. doi: 10.1371/journal.pdig.0001008 (PMC12445462; doi:10.1371/journal.pdig.0001008)
Supplement: S12 Table — AUPRC 95% confidence intervals are calculated by bootstrapping. (S12_Table.DOCX) [file pdig.0001008.s012.docx]

**S12 Table: AUPRC for models using structured data only.** AUPRC 95% confidence intervals are calculated by bootstrapping.

| **Structured Data** | **Elastic Net** | **Random Forest** | **XGBoost** |
| --- | --- | --- | --- |
| Baseline | 0.610 (0.594-0.625) | – | – |
| EHR | 0.593 (0.57-0.616) | 0.613 (0.591-0.636) | 0.626 (0.604-0.649) |
| EHR + ADI | 0.593 (0.57-0.615) | 0.614 (0.591-0.636) | 0.629 (0.607-0.651) |
| EHR + ACS | 0.596 (0.574-0.619) | 0.612 (0.59-0.634) | 0.629 (0.607-0.651) |
| EHR + ADI + ACS | 0.596 (0.573-0.618) | 0.614 (0.592-0.636) | 0.629 (0.607-0.651) |
| EHR + ADI + ACS + EMS | 0.603 (0.581-0.625) | 0.623 (0.601-0.644) | 0.640 (0.619-0.661) |
