# Supplementary material for: Integrating multiple data sources to predict all-cause readmission or mortality in patients with substance misuse
Source: PLOS Digit Health. 2025 Sep 18;4(9):e0001008. doi: 10.1371/journal.pdig.0001008 (PMC12445462; doi:10.1371/journal.pdig.0001008)
Supplement: S13 Table — AUPRC 95% confidence intervals are calculated by bootstrapping. (S13_Table.DOCX) [file pdig.0001008.s013.docx]

**S13 Table: AUPRC for multimodal prediction models compared to models that used structured data only.** AUPRC 95% confidence intervals are calculated by bootstrapping.

| **Data and Model Type** | **AUPRC (95% CI)** |
| --- | --- |
| Structured Data  **XGBoost** | 0.640 (0.619-0.661) |
| Structured + Unstructured Data **(BoW) XGBoost** | 0.640 (0.616-0.663) |
| Structured + Unstructured Data **(SapBERT)** **XGBoost** | 0.637 (0.613-0.661) |
| Structured + Unstructured Data **(SapBERT)** **Early Fusion** **Deep Learning** | 0.619 (0.596-0.643) |
| Structured + Unstructured Data **(SapBERT)** **Joint Fusion** **Deep Learning** | 0.578 (0.555-0.602) |
| Structured + Unstructured Data **(SapBERT) Late Fusion Deep Learning** | 0.638 (0.616-0.660) |
