# Supplementary material for: Integrating multiple data sources to predict all-cause readmission or mortality in patients with substance misuse
Source: PLOS Digit Health. 2025 Sep 18;4(9):e0001008. doi: 10.1371/journal.pdig.0001008 (PMC12445462; doi:10.1371/journal.pdig.0001008)
Supplement: S14 Table — (S14_Table.DOCX) [file pdig.0001008.s014.docx]

**S14 Table: Sensitivity and specificity analysis.**

| **Specificity** | **Sensitivity** |
| --- | --- |
| XGBoost: EHR + ADI + ACS + EMS   \| **specificity** \| **sensitivity** \| **ppv** \| **npv** \| \| --- \| --- \| --- \| --- \| \| 0.1 \| 0.982 \| 0.385 \| 0.906 \| \| 0.2 \| 0.956 \| 0.407 \| 0.888 \| \| 0.3 \| 0.914 \| 0.428 \| 0.859 \| \| 0.4 \| 0.865 \| 0.453 \| 0.838 \| \| 0.5 \| 0.810 \| 0.481 \| 0.821 \| \| 0.6 \| 0.741 \| 0.515 \| 0.801 \| \| 0.7 \| 0.648 \| 0.553 \| 0.776 \| \| 0.8 \| 0.530 \| 0.603 \| 0.748 \| \| 0.9 \| 0.395 \| 0.693 \| 0.722 \| | XGBoost: EHR + ADI + ACS + EMS   \| **sensitivity** \| **specificity** \| **ppv** \| **npv** \| \| --- \| --- \| --- \| --- \| \| 0.1 \| 0.986 \| 0.800 \| 0.656 \| \| 0.2 \| 0.964 \| 0.760 \| 0.678 \| \| 0.3 \| 0.934 \| 0.722 \| 0.699 \| \| 0.4 \| 0.897 \| 0.690 \| 0.723 \| \| 0.5 \| 0.823 \| 0.618 \| 0.742 \| \| 0.6 \| 0.751 \| 0.580 \| 0.766 \| \| 0.7 \| 0.638 \| 0.526 \| 0.788 \| \| 0.8 \| 0.512 \| 0.485 \| 0.817 \| \| 0.9 \| 0.330 \| 0.435 \| 0.852 \| |
| Baseline: Age, Number of prior encounters within 30 days   \| **specificity** \| **sensitivity** \| **ppv** \| **npv** \| \| --- \| --- \| --- \| --- \| \| 0.1 \| 0.955 \| 0.379 \| 0.798 \| \| 0.2 \| 0.885 \| 0.388 \| 0.752 \| \| 0.3 \| 0.791 \| 0.395 \| 0.719 \| \| 0.4 \| 0.713 \| 0.405 \| 0.708 \| \| 0.5 \| 0.622 \| 0.417 \| 0.698 \| \| 0.6 \| 0.523 \| 0.430 \| 0.688 \| \| 0.7 \| 0.436 \| 0.450 \| 0.682 \| \| 0.8 \| 0.341 \| 0.499 \| 0.680 \| \| 0.9 \| 0.276 \| 0.613 \| 0.684 \| | Baseline: Age, Number of prior encounters within 30 days   \| **sensitivity** \| **specificity** \| **ppv** \| **npv** \| \| --- \| --- \| --- \| --- \| \| 0.1 \| 0.982 \| 0.763 \| 0.656 \| \| 0.2 \| 0.942 \| 0.663 \| 0.672 \| \| 0.3 \| 0.871 \| 0.572 \| 0.685 \| \| 0.4 \| 0.739 \| 0.466 \| 0.681 \| \| 0.5 \| 0.619 \| 0.432 \| 0.686 \| \| 0.6 \| 0.516 \| 0.417 \| 0.694 \| \| 0.7 \| 0.417 \| 0.408 \| 0.708 \| \| 0.8 \| 0.288 \| 0.393 \| 0.720 \| \| 0.9 \| 0.181 \| 0.386 \| 0.758 \| |
