# Supplementary material for: Integrating multiple data sources to predict all-cause readmission or mortality in patients with substance misuse
Source: PLOS Digit Health. 2025 Sep 18;4(9):e0001008. doi: 10.1371/journal.pdig.0001008 (PMC12445462; doi:10.1371/journal.pdig.0001008)
Supplement: S15 Table — *Other refers to not alcohol and not opiate. (S15_Table.DOCX) [file pdig.0001008.s015.docx]

**S15 Table: Structured data-only XGBoost Model performance by substance type. ***Other refers to not alcohol and not opiate.

| **Substance** | **AUC**  **(95% CI)** | **Specificity**  **(95%CI)** | **Sensitivity**  **(95CI)** |
| --- | --- | --- | --- |
| Any, n= 5,609: | 0.746  (0.732-0.759) | 0.662  (0.651-0.791) | 0.709  (0.577-0.727) |
| Alcohol, n = 2,878: | 0.768  (0.751-0.786) | 0.747  (0.678-0.760) | 0.657  (0.592-0.727) |
| Opiate, n = 2,098: | 0.704  (0.680-0.727) | 0.647  (0.626-0.749) | 0.679  (0.572-0.710) |
| *Other, n = 633: | 0.764  (0.726-0.801) | 0.626  (0.570-0.844) | 0.781  (0.563-0.840) |
