# Supplementary material for: Integrating multiple data sources to predict all-cause readmission or mortality in patients with substance misuse
Source: PLOS Digit Health. 2025 Sep 18;4(9):e0001008. doi: 10.1371/journal.pdig.0001008 (PMC12445462; doi:10.1371/journal.pdig.0001008)
Supplement: S1 Fig — (a) The early-fusion approach concatenated variables from the structured-only data sources with the convolutional layer output to which the SapBERT-based embeddings are fed. (b) The joint-fusion approach combined a dense layer representation of structured features with CNN-based embedding of clinical text. (c) The late fusion approach combined predictions from an individually trained XGB model (created from structured data) and a CNN model (created from text-based features) and fed them into a logistic regression (LR) model to make the final prediction. (S1_Fig.DOCX) [file pdig.0001008.s016.docx]

**S1 Fig: Illustration of our deep learning multimodal architectures.** (a) The early-fusion approach concatenated variables from the structured-only data sources with the convolutional layer output to which the SapBERT-based embeddings are fed. (b) The joint-fusion approach combined a dense layer representation of structured features with CNN-based embedding of clinical text. (c) The late fusion approach combined predictions from an individually trained XGB model (created from structured data) and a CNN model (created from text-based features) and fed them into a logistic regression (LR) model to make the final prediction.

(a)

(b)

(c)
